# Evaluating the diagnosis and treatment of *Chlamydia trachomatis* and *Neisseria gonorrhoeae* in pregnant women to prevent adverse neonatal consequences in Gaborone, Botswana: protocol for the Maduo study

**DOI:** 10.1186/s12879-022-07093-z

**Published:** 2022-03-07

**Authors:** Adriane Wynn, Aamirah Mussa, Rebecca Ryan, Emily Hansman, Selebaleng Simon, Bame Bame, Badani Moreri-Ntshabele, Doreen Ramogola-Masire, Jeffrey D. Klausner, Chelsea Morroni

**Affiliations:** 1 University of California, San Diego, USA; 2 Botswana Sexual and Reproductive Health Initiative, Gaborone, Botswana; 3 Botswana-Harvard AIDS Institute Partnership, Gaborone, Botswana; 4 University of California, Los Angeles, USA; 5 University of Botswana, Gaborone, Botswana; 6 University of Southern California, Los Angeles, USA; 7 University of Edinburgh, Edinburgh, UK

**Keywords:** Sexually transmitted infection, Pregnancy, Neonatal outcomes, Cluster controlled trial, Botswana

## Abstract

**Background:**

*Chlamydia trachomatis* (CT) and *Neisseria gonorrhoeae* (NG) are extremely common sexually transmitted infections (STIs) that are associated with adverse birth and neonatal outcomes, and the risk of vertical transmission of CT and NG during delivery is high. The majority of CT and NG infections are asymptomatic and missed by the standard of care in most countries (treatment based on symptoms). Thus, it is likely that missed maternal CT and NG infections contribute to preventable adverse health outcomes among women and children globally. This study aims to assess the effectiveness of CT and NG testing for asymptomatic pregnant women to prevent adverse neonatal outcomes, understand the inflammatory response linking CT and NG infections to adverse neonatal outcomes, and conduct an economic analysis of the CT and NG testing intervention.

**Methods:**

The Maduo (“results” in Setswana) is a prospective, cluster-controlled trial in Gaborone, Botswana to compare a near point-of-care CT and NG testing and treatment intervention implemented in “study clinics” with standard antenatal care (World Health Organization-endorsed “syndromic management” strategy based on signs and symptoms without laboratory confirmation) implemented in “standard of care clinics” among asymptomatic pregnant women. The primary outcome is vertical transmission of CT/NG infection. Secondary outcomes include preterm birth (delivery < 37 completed weeks of gestation) and/or low birth weight (< 2500 g). The trial will also evaluate immunological and inflammatory markers of adverse neonatal outcomes, as well as the costs and cost-effectiveness of the intervention compared with standard care.

**Discussion:**

The Maduo study will improve our understanding of the effectiveness and cost-effectiveness of CT and NG testing among asymptomatic pregnant women. It will also increase knowledge about the CT/NG-related immune responses that might drive adverse neonatal outcomes. Further, results from this study could encourage expansion of STI testing during antenatal care in low resource settings and improve maternal and neonatal health globally.

*Trial registration*: This trial is registered with ClinicalTrials.gov (Identifier NCT04955717, First posted: July 9, 2021)).

## Background

According to the World Health Organization (WHO), *Chlamydia trachomatis* (CT) and *Neisseria gonorrhoeae* (NG) are extremely common sexually transmitted infections (STIs), with 127 million (95% UI: 95.1–165.9 million) and 86.9 million (95% UI: 58.6–123.4 million) cases respectively worldwide in 2016 [1]. In 2012, approximately 90% of STIs occurred in low and middle-income countries (LMICs) [2] and sub-Saharan Africa was identified as the WHO region with highest STI incidence and prevalence [3]. Women are particularly vulnerable to STIs due to increased susceptibility to genital tract infections compared to men [4, 5], gender inequalities that can result in decreased power to negotiate sex or condom use [6], and increased risk for STI-related adverse health outcomes, including pelvic inflammatory disease and sequelae [7–9]. Recent analysis using the Spectrum-STI model in South Africa found that women had a higher prevalence of CT (14.7% vs. 6.0%) and NG (6.6% vs. 3.5%) compared to men [10] and prevalence estimates are higher among pregnant women [11]. While research is limited in our study setting of Botswana, a small study among patients seeking HIV care found that women were more likely to be infected with CT and/or NG compared to men (7% vs. 1%) [12].

There is evidence that maternal CT and NG infections are associated with adverse birth and neonatal outcomes [13], including preterm birth and low birth weight [14–18], miscarriage [14, 17], and stillbirth [14, 19]. The risk of vertical transmission of CT and NG during delivery is about 50% [20]. Among infants born to mothers with untreated CT infection, 30–50% develop clinical conjunctivitis and 10–20% develop CT-related pneumonia [20, 21]. Among infants born to mothers with untreated NG infection, the risk of conjunctival infection is up to 48%, which can result in corneal damage and blindness [22]. A systematic review and meta-analysis found that maternal NG infections increased the risk of preterm birth (OR 1.55, 95% CI 1.21–1.99); premature rupture of membranes (OR 1.41, 95% CI 1.02–1.92); perinatal mortality (OR 2.16, 95% CI 1.35–3.46); low birth weight (OR 1.66, 95% CI 1.12–2.48); and ophthalmia neonatorum (OR 4.21, 95% CI 1.36–13.04) [23]. A recent study in South Africa found that among infants born to a mother with CT, NG, and/or *Trichomonas vaginalis* infections, 37% had nasopharyngeal colonization with the same STI organism [24]. Further, HIV-infected pregnant women with CT and/or NG infections were found to have an increased risk of vertical transmission of HIV [25, 26]. Other studies involving CT and NG infections in neonates are over 20 years old [22, 27, 28].

Pregnancy is an immunologically dynamic state, and immunological changes associated with STIs likely contribute to adverse neonatal outcomes [29, 30]. Inflammation caused by intrauterine infection may account for 25–40% of preterm births [31, 32]. Two systematic reviews found strong associations between cervical infections, such as CT, and local inflammation that may lead to premature labor and preterm birth [28, 33–36]. CT may also infect fetal membranes, triggering a harmful inflammatory response with cytokine release leading to miscarriage or preterm labor [37–40]. However, many gaps exist in our understanding of how CT/NG-related immune responses might drive adverse neonatal outcomes, such as preterm birth and low birth weight [41–47].

In most LMICs, syndromic management is used to identify and manage STIs other than HIV and syphilis. Syndromic management utilizes an algorithm to classify symptoms and clinical signs into STI syndromes, and patients are treated with standardized drug regimens [48, 49]. The WHO guidelines provide algorithms for six syndromes, including urethral discharge, vaginal discharge, genital ulcers, scrotal swelling, lower abdominal pain, and neonatal conjunctivitis [50]. Syndromic management has advantages, including timely treatment, limited training requirements, no need for laboratory resources, and low costs [51]; however, the syndromic approach has many draw backs. It lacks specificity, causing unnecessary exposure to antibiotics. It also has low sensitivity, missing the majority of infections, which are asymptomatic [52–55]. Several studies have shown poor sensitivity because women with CT or NG often have no symptoms [56]. A systematic review and meta-analysis of the performance of abnormal vaginal discharge flowcharts found that diagnostic performance was low, regardless of whether a risk assessment or clinical examination was included or the flow chart was WHO- or locally developed [57]. In Botswana, previous research among pregnant women found that syndromic management was no better than flipping a coin for treating CT and NG infections [58].

In Botswana and most countries, symptomatic infections among pregnant women are treated with standardized drug regimens [59]; however, asymptomatic infections are missed. Research has found that asymptomatic infections also cause adverse health outcomes [51, 60]. A Swedish study found that, among 109 asymptomatic adolescent girls with untreated, culture-proven CT infection, 3.7% reported being hospitalized for salpingitis or seen in the emergency department for lower abdominal pain and vaginal discharge over a 3-month observation period [61]. Another study that included 20 women coinfected with CT and NG who received adequate therapy for gonococcal but not chlamydial infection, found that 30% of participants received a diagnosis of PID after seven weeks of follow-up [62]. A study among female sex workers in South Africa found that cervicovaginal inflammatory markers were elevated in women with both symptomatic and asymptomatic STIs [63]. Therefore, women with asymptomatic STIs had subclinical inflammation, which may increase their susceptibility to HIV infection [64].

While etiologic testing has been proven to be superior to syndromic management in terms of identifying and treating STIs [65, 66], and reducing PID [67], there is no consensus as to whether antenatal testing and treatment for CT and NG prevents adverse birth and neonatal outcomes as research results are mixed [68]. In the United States, the Centers for Disease Control and Prevention (CDC) recommends CT and NG testing for all pregnant women less than 25 years of age and older pregnant women who are at increased risk (e.g., new or multiple sex partners, exchanging sex for payment, illicit drug use, or a history of STIs) [69]. However, the US Preventive Services Task Force has recently called for more research with evidence on the effectiveness of screening pregnant persons [70]. As accurate and rapid testing technology is increasingly affordable, more evidence on the benefits of antenatal testing for curable STIs is needed.

The proposed study will estimate the prevalence and correlates of CT and NG infection among pregnant women who present to antenatal care (ANC) without STI-related symptoms, and thus would not be treated for infections through standard antenatal care. We will assess the impact of antenatal CT/NG testing to prevent vertical transmission and adverse birth and neonatal health outcomes compared to the standard of care. We will explore the inflammatory response to CT/NG infections and associations with vertical transmission through markers of tissue inflammation specific to the Xpert^®^ as well as plasma inflammatory signatures. We will also estimate the total cost, unit costs, and cost-effectiveness of CT/NG infection testing among asymptomatic pregnant women compared to syndromic management. This study will take place in Botswana, a country with the second highest HIV prevalence in the world, including a 33.3% prevalence among pregnant women. [71]. The specific aims for the Maduo (“results” in Setswana) study are:

1. To determine the burden of CT and NG infections and correlates of infection among asymptomatic pregnant women in Gaborone, Botswana by a) using diagnostic testing to estimate the prevalence of CT and NG infections at three time points (first ANC visit, third trimester ANC visit, and postnatal visit) and estimating the incidence of infections between those visits and (b) assessing the correlates of infection.
2. To compare longitudinal neonatal outcomes for pregnant women tested for CT and NG infections during antenatal care with women who received standard antenatal care by (a) estimating the frequency of vertical transmission of CT and NG infections and neonatal outcomes and the association with antenatal testing and treatment; and (b) assessing independent factors that may be predictive of adverse neonatal outcomes.
3. To assess markers of inflammatory response to CT and NG infection during pregnancy and associations with vertical transmission of CT and NG, preterm birth, and low birth weight by a) identifying immune/inflammatory signatures associated with vertical transmission of CT and NG, preterm birth and low birth weight; b) determining the association between the Xpert^®^ CT/NG assay’s pathogen-specific cycle threshold value (Ct) and CT/NG transmission; and c) evaluating the frequency and distribution of Sample Adequacy Control (SAC) cycle threshold values (Ct) from the Xpert^®^ CT/NG assay, and evaluate any correlations with the transmission of CT/NG to neonates.
4. To determine the total cost of the antenatal testing and treatment intervention as well as the costs per woman tested, treated, and cured and the cost per any adverse health outcome averted.

## Methods/design

### Setting

The Maduo study will be conducted in up to eight public antenatal clinics run by the Greater Gaborone District Health Management Team (DHMT) in Botswana. Botswana national guidelines recommend that women routinely attend a minimum of five antenatal clinic visits after the booking visit (at 16–20, 24–28, 32, 34–36 and 38–40 weeks of gestation). The first ANC visit typically includes the following services as part of standard care:

- Assessments of obstetric and general medical history with an internal pelvic examination performed by a midwife if clinically indicated.
- Provider-initiated counselling and testing for HIV and syphilis infection.
  - HIV rapid testing is conducted at the first ANC visit and all HIV negative pregnant women receive repeat HIV testing every three months with documented results in the third trimester, at delivery, and 6 weeks post-delivery. Women who test positive for HIV infection receive a second rapid HIV test, and, if positive, are referred for specialist review and initiation of antiretroviral therapy [72].
  - Syphilis screening is conducted using rapid plasma reagin (RPR) at the first antenatal visit and repeated at week 34–36 gestation. Women with a reactive RPR test are treated with benzathine penicillin once weekly for three consecutive weeks (or with azithromycin/erythromycin for those with a penicillin allergy) [73].
- Other routine testing includes hemoglobin, Rhesus factor, and urinalysis (protein and glucose testing).
- Iron and folate supplementation and tetanus toxoid immunization are provided.
- Syndromic management for STIs is provided for women presenting with genital symptoms. Women who meet the criteria for syndromic management (for example, women reporting vaginal discharge with cervical mucus on examination meet the criteria for ‘vaginal discharge syndrome’) will be treated with antimicrobials according to the most appropriate treatment algorithm.
- A summary of the clinical findings, including investigation results and treatments provided, are documented in appropriate sections of the patient hand-held Botswana Obstetric Record by the clinic midwife.

### Study design

This is a prospective, two-arm, non-randomized, controlled cluster trial. Participants in sites designated as testing clinics will receive the antenatal CT and NG testing and treatment intervention and those in designated standard-of-care clinics will receive the standard antenatal care. All participants will receive intimate partner violence (IPV) and perinatal depression screening with linkages to services as well as postnatal CT and NG testing and treatment.

### Selection of clinics

The clinics were selected in consultation with DHMT administrators and clinic staff, based on the availability of a secure and private space for near point-of-care STI testing, the average numbers of new ANC patients per month, and if clinic staff were interested in participating in the study. Based on clinic data from the last six months, selected clinics had an average of 40–74 new ANC patients per month. Site assessments indicated that each clinic could enroll around 7–10 eligible women per week, taking 25–35 weeks to recruit our required sample of 250 women per study arm. All DHMT clinics are government-run, free at the point of use, and provide clinical antenatal care according to national Botswana clinical guidelines described above.

### Study implementation team

The study will be managed by a Study Coordinator who will oversee the Nurse Coordinator and all staff. A Nurse Coordinator will be based at study clinics, supervising a team consisting of four experienced research assistants who are responsible for day-to-day conduct of study procedures including recruitment, enrollment and follow-up. A study clinician will also be on call for clinical questions from the study team, to dispense treatment and for IPV and depression referrals. The co-Principal Investigators are responsible for the overall conduct and management of the study.

### Study participants and eligibility criteria

Pregnant women who are 15 years of age or older will be invited to join the study if they meet the criteria outlined in Table 1.

**Table 1:** Inclusion and exclusion criteria for the Maduo study, Gaborone, Botswana, 2019–2022

Inclusion criteria	Exclusion criteria
Criteria assessed during vitals collection	
Age ≥ 15 years	Unable to give informed consent
Pregnant	
First antenatal care visit	
Intention to reside in Gaborone through postnatal care	
Criteria assessed after being seen by healthcare provider	
27 weeks, 0 days of gestation or less (confirmed by checking patient-held obstetric record or using a gestational wheel if absent from obstetric record)	
Not treated for an STI in the last 30 days (including recruitment date)	
Willingness to self-collect vaginal specimens for *Chlamydia trachomatis/Neisseria gonorrhoeae* testing	
Willingness to have an ocular and nasopharyngeal specimen collected from newborn for *Chlamydia trachomatis/Neisseria gonorrhoeae* testing	

### Recruitment and informed consent

Upon arrival at the participating antenatal clinics, women will be provided with general information about the study though a 5-minute waiting room talk, provided by a member of the research team. At the testing clinic, women will receive an overview of antenatal testing and treatment and, at both clinics, women will receive an overview of postnatal and infant testing and treatment.

**Fig. 1:**
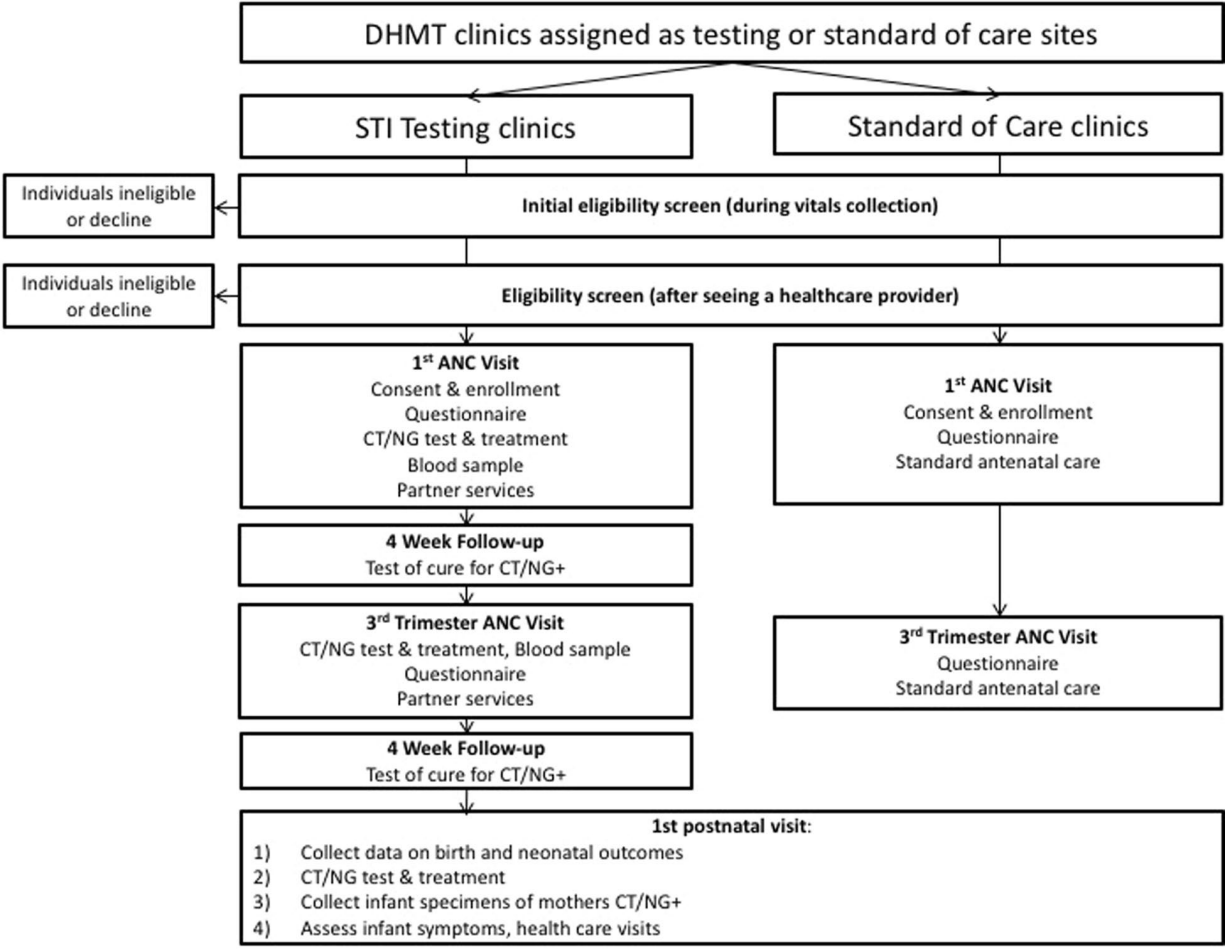
Study flow for the Maduo study, Gaborone, Botswana, 2019–2022

Participants will be assessed for eligibility based on the algorithm found in Fig. 1. Initial eligibility criteria will be assessed by research assistants in the vitals collection room. Those who are preliminarily eligible will be asked to return to study staff after their visit with the healthcare provider for a second screening and if eligible, offered informed consent and enrollment. Women who have been clinically diagnosed through syndromic management as having an STI-related syndrome (e.g. abnormal vaginal discharge syndrome) and prescribed treatment within the last 30 days will be provided additional information about STIs and partner notification but will not be eligible for participation in the study.

Prior to enrollment, all participants will be required to complete written informed consent. Information and consent procedures will be conducted in Setswana or English, as preferred by the individual participant. Women who are unable to read and/or write will be required to have an impartial witness present during the above procedures. Women aged 15–17 years of age will be eligible to participate with verbal parental consent. Parents or guardians of interested 15–17-year olds will be notified if their child is interested in participating and verbal consent will be obtained over the phone and documented.

### Intervention summary

As shown in Fig. 1, all participants will respond to an interviewer-administered questionnaire at the baseline (first ANC) visit, during a third trimester visit (30–36 weeks gestation), and at the first postnatal care visit (6–8 weeks postpartum). All women will provide a self-collected vaginal specimen for CT/NG testing at their first postnatal care visit and will be provided with results and treatment within 24 h of testing. Infants of mothers who test positive will have an ocular and nasopharyngeal swab collected for CT/NG testing and treatment.

Women enrolled in the testing clinics will provide self-collected vaginal specimens for CT/NG testing at the baseline and third trimester visits, and will be provided with results and treatment within 24 hours of testing. Women who test positive for CT/NG will also provide a self-collected vaginal specimen at a test-of-cure visit four weeks after they receive treatment and they will be re-treated, if necessary. Participants in the testing clinic will also be asked to provide 2–4 mL of blood at enrollment and at the third trimester ANC visit for immune/inflammation analysis.

### Baseline procedures

#### All clinics

After participants have been seen by a healthcare provider, met eligibility criteria, and provided informed consent, an interviewer-administered questionnaire will be conducted in the participant’s preferred language (English or Setswana). The questionnaire will collect: (1) socio-demographic characteristics; (2) sexual history; (3) antibiotic usage; (4) other risk factors, including depression (Edinburgh Postnatal Depression Scale) [74], alcohol use (AUDIT-C) [75], and any smoking during pregnancy; and (5) partner characteristics; and (6) intimate partner violence experience (Conflict Tactics Scale) [76]. Additional information will be abstracted from the patient-held obstetric record, including: (1) obstetric history, (2) HIV status, including date of diagnosis, most recent CD4 cell and viral load counts and dates; (3) ART initiation; and (4) other maternal conditions (e.g., hypertension). Table 2 shows the measures to be collected, definitions, timing, and sources. Questionnaire responses are self-reported. Record abstraction will start with the patient obstetric records and, in the case of missing information, the facility record will be consulted. A transabdominal obstetric ultrasound examination to estimate gestational age will be performed by a trained practitioner (doctor or midwife) on a sub-sample of participants to validate gestational age measurement via obstetric record abstraction. Participants’ locator information, including home and work address, telephone number, and contact number for a trusted person (if the participant wishes to provide) will be collected to facilitate follow-up and provision of results. Locator information will be kept separately from de-identified electronic data to protect confidentiality.

**Table 2:** Summary of data collection for the Maduo study, Gaborone Botswana, 2019–2022

Category	Measure	Definition	Time point	Source
Adverse neonatal outcomes	Infant CT/NG infection	Positive for CT/NG using the GeneXpert and infant ocular and nasopharyngeal specimens	Postnatal	Study records
	Infant conjunctivitis	Red or swollen eyelids, discharge from the eyes	Postnatal	Patient held and clinic obstetric record
	Infant pneumonia	Cough or difficult breathing plus at least one of the following: Fast breathing Lower chest wall indrawing In addition, either crackles or pleural rub may be present on chest auscultation	Postnatal	Patient held and clinic obstetric record
	Preterm delivery	Delivery < 37 weeks, 0 days gestational age	Postnatal	Patient held and clinic obstetric record
	Low birth weight	< 2500 g	Postnatal	Patient held and clinic obstetric record
CT/NG Treatment group	Care in testing clinic Care in SoC clinic	Enrolled in either the testing or SoC clinic at baseline	Baseline	Study records
HIV Status	HIV Negative HIV Positive Known Positive Newly Diagnosed	HIV negative if never diagnosed with HIV prior to or during pregnancy HIV positive if a positive diagnosis was received prior to or during pregnancy. We will conduct a sub analysis among women with a known (prior to conception) and new (during pregnancy) HIV diagnosis	Baseline, 3rd trimester, postnatal	Patient held and clinic obstetric record
Antiretroviral history	Not on ART On ART Initiated during pregnancy Initiated prior to conception	Not on ART if not initiated or discontinued during pregnancy On ART if ART use reported at baseline, third trimester and postnatal care ART duration measured as initiated during pregnancy or initiated prior to conception. We will also conduct a sub-analysis among women on ART for < 2, 2–5, or > 5 years	Baseline, 3rd trimester, postnatal	Patient held and clinic obstetric record
Viral Suppression	Virally suppressed Not virally suppressed	Virally suppressed when HIV-1 RNA < 400 copies/mL at all time points Not virally suppressed when HIV-1 RNA ≥ 400 copies/mL at any time point	Baseline, 3rd trimester, postnatal	Patient held and clinic obstetric record
Potential maternal confounding factors	Maternal socio-demographics	Age, education, employment status, income level	Baseline	Questionnaire (self-report)
	Obstetric history	Parity, number of previous miscarriages, preterm birth, low birth weight infants	Baseline	Patient held record and participant self-report
	Other maternal conditions and treatments	Urinary tract infection, syphilis diagnosis, genital ulcer, gestational diabetes (fasting glucose ≥ 92 mg/dL, 1-h glucose ≥ 180 mg/dL, or 2-h glucose ≥ 153 mg/dL), anemia (hemoglobin ≤ 10 g/dL, gestational hypertension (at least one blood pressure elevation of systolic blood pressure ≥ 140 mmHg or diastolic blood pressure ≥ 90 mmHg after 20 weeks pregnancy), body mass index, SARS-COV-19 infection	Baseline, 3rd trimester, postnatal	Patient held and clinic obstetric record
	Antibiotic usage during pregnancy	Azithromycin, Erythromycin, Doxycycline, Metronidazole, Ceftriaxone	Baseline, 3rd trimester, postnatal	Patient held and clinic obstetric record
	Other risk factors	Maternal alcohol use (AUDIT-C), smoking (any during pregnancy)	Baseline, 3^rd^ trimester, postnatal	Questionnaire (self-report)
	Partner characteristics	Number of current partners, length of relationships, marital status, children, intimate partner violence (Conflict Tactics Scale)	Baseline, 3rd trimester, postnatal	Questionnaire (self-report)
	Partner treatment	Partner treatment outcomes and timing of treatment	Baseline, 3rd trimester, postnatal	Questionnaire (self-report)
Healthcare utilization	Routine ANC	Number of ANC visits between first ANC and third trimester and deliver	3^rd^ trimester, postnatal	Patient held and clinic obstetric record
	Antenatal admissions	Number of inpatient admissions and length of stay between baseline and postnatal care	3^rd^ trimester, postnatal	Patient held and clinic obstetric record
	Infant admissions	Number of inpatient admissions and length of stay	Postnatal, immunization	Patient held and clinic obstetric record
	Infant interventions	Emergency transportation by ambulance Medication, radiology, laboratory, and respiratory care services	Postnatal, immunization	Patient held and clinic obstetric record

Intimate partner violence and perinatal depression will be proactively assessed and managed for all participants. Women who report IPV or have an Edinburgh Postnatal Depression Scale score of ≥ 9 or answer affirmative to question 10 (“the thought of harming myself has occurred to me”) will be assessed by a study clinician and, where appropriate, referred to local support services, such as the Postnatal Mental Health Society of Botswana and the Botswana Gender Based Violence Support and Prevention Centre. We will also provide a 24-hour emergency contact line through which participants can receive information and support and referrals in general.

#### Testing clinics

Women in the testing clinics will additionally provide one self-collected vaginal swab specimen for CT/NG testing. Study staff will use a laminated pictorial guide and a demonstration kit to explain the correct way to collect vaginal specimens. Participants will be given the opportunity to ask questions and to clarify areas of misunderstanding. Participants will collect the vaginal specimens in a private room with a lock, or in the clinic bathroom. Specimens for CT/NG testing will be processed on site and participants will be provided results on the same day as specimen collection (in person or by phone). If the participant is initially unreachable, up to ten contact attempts will be made on different days and times. Thereafter, study staff will use the alternative contact numbers provided by the participant to request that she contacts the clinic. Participant results and the nature of the test will never be disclosed to anyone other than the participant herself.

Participants testing positive for CT/NG will be treated using WHO recommended treatments and will receive azithromycin 1 g by mouth for CT and ceftriaxone 500 mg by intramuscular injection for NG. Participants who test positive will also be provided with counselling on the importance of partner notification and treatment and abstinence from sexual intercourse until seven days after both the participant and partner has been treated.

Participants will be given several options for partner services: (1) No disclosure, in the case of risk for intimate partner violence; (2) participant-led disclosure, facilitated by a contact slip notifying the partner that they are a contact of an STI (3) supportive disclosure, where the participant is assisted by trained study staff either in person or over the phone in notifying their partners, and given the option of bringing partners to the study clinics for counselling and treatment; (4) expedited partner therapy (EPT), where the participant can bring treatment to partners prior to partner examination by a healthcare provider. In the case of EPT for NG infection, partners will be given 800 mg cefixime orally in a single dose. Women and their partners will be advised to abstain from sexual intercourse for seven days from the day of treatment to reduce the risk of reinfection. All study staff will be trained on appropriate and effective methods for counselling women on how to communicate test results to partner(s) in a nonthreatening and blameless manner. For example, it is important to clarify that the duration of CT/NG infections during pregnancy is unknown, many infections are asymptomatic, and infections do not have implications about partner fidelity.

Additionally, participants who test positive for CT and/or NG will be asked to attend a ‘Test of cure’ visit four weeks after completion of treatment for a repeat self-sample vaginal swab to be tested for CT and NG*.* If the test of cure is positive, participants will be re-treated and provided with further counselling about STIs and the importance of partner notification, treatment and abstinence from sexual intercourse until seven days after both the participant and partner have been treated.

Participants with a negative test for CT or NG, but with clinical evidence of cervicitis or vulvovaginitis based on symptom history and clinical examination will be treated with STI syndromic management as per national guidelines. Any treatment provided will be clearly documented, both on the REDCap electronic case report form (CRF) and in the participant’s handheld medical record. If the test result is invalid or indeterminate, women will be asked to provide an additional self-collected swab and will be given further counselling on self-collection, to allow for repeat testing.

Participants in the testing clinics will also provide 2–4 mL of peripheral blood, which will be collected by the study nurse or phlebotomy-trained research assistant using standard blood draw procedures. After collection, the blood will be mixed and stored at 4 °C in a study cooler. The study driver will transport the blood specimen to the lab, where a lab technician will centrifuge blood samples within eight hours after collection. Samples will be stored at 80 °C until transport in dry-ice to the Human Immune Monitoring Center at Stanford University. Using the Luminex xMAP^®^ multiplex assay method, plasma will be screened for the presence of more than 45 immunologically active proteins involved in key biological processes, including response to infections, chemokines, inflammatory response, and cytokine-mediated signaling pathways. Importantly, the panel includes previously identified cytokines associated with adverse neonatal outcomes, including IL-1a, IL-1b, IL-2, IL-4, IL-6, IL-8, IL-10, TNF-a, IFN-y, MIP-1a, MIP-1b, RANTES, GM-CSF, and MCP-1.

### Third trimester

#### All clinics

Following enrollment, all participants will receive routine antenatal care according to the national guidelines, as previously described. Thereafter, all participants will be asked to attend another study visit during a routine ANC visit during their third trimester (at 30–36 weeks’ gestation). To prevent loss to follow-up, women will be called by the study team at 30 weeks’ gestation to schedule the third trimester visit. Participants will also receive a reminder call on the day before their scheduled visit. A digital study calendar will be used to track reminder calls and follow-up appointments.

All participants will respond to an interviewer-administered questionnaire to obtain information on mutable characteristics, such as risk factors for adverse birth and neonatal outcomes (e.g. smoking, alcohol use, IPV experience, depression risk, and COVID-19-related stress). The patient held obstetric record will also be abstracted for new diagnoses (e.g. HIV, hypertension, COVID-19) and care utilization (e.g. number of ANC visits).

#### Testing clinics

Participants will provide one self-collected vaginal swab for CT/NG testing using the GeneXpert platform. Specimens for CT/NG testing will be processed and participants will be given their results on the same day as specimen collection. Those who test positive will be treated, counselled on partner notification and treatment, and asked to attend a test-of-cure visit four weeks after treatment completion, in the same manner as the baseline visit. An additional blood specimen will be collected for plasma separation, storage, and shipping for immune/inflammation analysis.

#### Postnatal care

All participants will be asked to attend a postnatal follow-up study visit approximately 6–8 weeks after delivery, and all participants will receive CT and NG testing as described at baseline. Study staff will collect information on key birth and neonatal outcomes and serious events from the obstetric records. Data on delivery and neonatal characteristics will be abstracted from the post-delivery discharge summary including date, time, and location of birth; delivery method; gestational age; and birth weight (Table 2). Participants with an adverse outcome e.g. miscarriage, stillbirth or neonatal death will be contacted by telephone and, if they consent to continue in the study, will be asked to attend the study clinic for their follow-up visit on a day when no other post-partum mothers are present in clinic, to prevent distress. Clinical questions about the neonate will be omitted from the questionnaire for these women. Participants reporting health concerns that require further clinical management will be referred to local health services, as indicated.

To prevent loss to follow-up, women will be called by the study team at 3 weeks post delivery to schedule the postnatal visit. Participants will also receive a reminder call on the day before their scheduled visit. A digital study calendar will be used to track reminder calls and follow-up appointments.

#### Neonatal care

Neonates of women who test positive for CT and/or NG at the postnatal visit (6–8 weeks after delivery) will have an ocular swab and a nasopharyngeal specimen collected by the study clinician for CT/NG testing using the Xpert [77, 78]. Neonates with a positive test for CT and/or NG will be provided with intramuscular ceftriaxone 25–50 mg/kg as a single dose for neonatal gonorrhoea treatment and/or oral erythromycin 50 mg/kg/day in four divided doses per day for 14 days for neonatal chlamydia treatment in accordance with Botswana national guidelines. Neonates will also be linked with appropriate clinical services or follow-up care. The neonate’s clinical records will also be reviewed to ascertain whether the neonate had received prophylaxis or treatment for presumed neonatal chlamydia or gonococcal infection prior to the study visit. Neonates who are clinically unwell at the study visit will be assessed by the study doctor and referred for appropriate clinical management.

## Data collection procedures and measures

Collected data will be recorded directly into REDCap using a hand-held electronic device. At the enrollment visit, data will be entered into a study-specific CRF and clinical data form. Sociodemographic and behavioral information will be collected through participant self-report. For clinical data, study staff will review the participant’s Obstetric Record and hand-held medical records. Relevant information and measurements that were collected and recorded by the clinic midwife during the ANC visit will be added to the electronic clinical data form. Medical records will also be checked to clarify the participant’s medical history, including details of recent visits to a health center and any treatment the participant received. Aside from recording CT and NG test results and treatment, the data collection schedule, procedures, and instruments will be the same in all clinics.

For the cost analysis, both economic and financial costs associated with delivering STI testing and treatment will be collected prospectively from a healthcare system perspective using in-country data sources and data collection spreadsheets and manuals. Expenditures will be classified into four categories: (1) training and start-up, (2) personnel, including fringe benefits, (3) recurring (including supplies and services); (4) capital (items valued at $100 or more that last more than 1 year). Given the short time frame, all costs will be converted to 2022 US dollars and will not be discounted. Recurrent Costs will be collected using micro-costing techniques, including a time and motion study (TAM) to identify the time, personnel (e.g. nurse), activities (e.g. sample collection, quality control), and supplies used (e.g. gloves, sample collection swab) in each strategy. The TAM observer will follow a detailed protocol with costing sheets. The costs associated with supply procurement and shipping will be obtained from Central Medical Supplies. Salaries will be provided by the Botswana Ministry of Health. Training & start-up costs will be collected through grant expenditures and will include rentals (e.g. room and projector), staff transport, training supplies (e.g. flipchart and stationery), daily per diem for trainees, and refreshments. Capital costs, including equipment previously procured will be obtained from the Ministry of Health. Costs of equipment not been previously procured (e.g. GeneXpert system) will be obtained from the manufacturer. All capital costs will be annualized over their expected useful life using a discount rate of 3%.

## Adverse events and safety reporting

Adverse events and unanticipated problems that occur to study participants will be monitored, reported and managed according to policies and procedures of the Botswana Ministry of Health Research and Development Committee and the Eunice Kennedy Shriver National Institute of Child Health and Human Development [79]. Serious adverse events (stillbirth, early neonatal death, spontaneous abortion/miscarriage, domestic violence requiring medical care) that are related to study procedures and are not expected as an outcome of the study, will be reported to IRBs by the PI within 24 h.

The Maduo Study is assessing the impact of antenatal CT/NG testing in asymptomatic pregnant women using approved and established STI assays. Women testing positive for CT/NG will be provided with antibiotics that are widely used in the treatment of CT/NG. No new investigational products will be used in this study.

## Analysis

### Primary outcome measure

- Vertical transmission of CT and/or NG: neonates of mothers who test positive for CT and/or NG infections will be tested using both ocular and nasopharyngeal specimens.

### Secondary outcome measures

- Pregnancy outcome.
  - Miscarriage, defined as pregnancy loss before the 20th week of gestation, will be identified through self-report by the participants, and the medical record.
- Perinatal outcome.
  - Stillbirth, defined as fetal death at or after 20 weeks gestation and prior to delivery, will be identified through self-report by the participants, and the medical record.
- Neonatal outcomes.
  - Preterm birth, defined as delivery prior to 37 weeks’ gestation, will be identified through the obstetric record.
  - Low birth weight, defined as birth weight of less than 2500 g, will be identified through the obstetric record.
  - Conjunctivitis, defined as the presence of swollen eyelids and pus in eyes, will be identified through the obstetric record and during the care visit by the follow-up nurse/midwife.
  - Pneumonia, defined as cough or difficult breathing plus at least one of the following: fast breathing or lower chest wall indrawing. In addition, either crackles or pleural rub may be present on chest auscultation.
  - We will also ask mothers about prior neonatal symptoms and review the obstetric record.
- Maternal outcomes.
  - CT and NG infections: the prevalence of maternal CT/NG infections will be assessed among participants in the testing group at baseline and during the third trimester.
  - The incidence of infections between baseline and follow-up in the testing group.
  - Individual cytokines and groups of cytokines associated with vertical transmission of CT/NG, preterm birth, and low birth weight.
- Economic outcomes.
  - The total cost of the CT/NG test and treatment intervention for asymptomatic pregnant women.
  - The costs per woman tested, treated, and cured; and the incremental costs per adverse neonatal outcome averted (e.g. neonatal CT/NG infections, preterm birth, and low birth weight) comparing the testing intervention with syndromic management.

### Statistical analysis

Prevalence of each STI will be measured as the proportion of individuals testing positive for a given STI divided by the number of individuals tested at each time point. Exact binomial 95% confidence intervals for prevalence will be estimated. Incident infections will be identified when a participant tests negative for an STI during her first ANC visit and positive for an STI during follow-up visits. Time at risk will be the length of time between the first test date and the positive test date. Correlates of CT/NG infections will be identified using both bivariate analyses and multivariable analyses.

Our primary outcome (vertical transmission of CT and NG) and secondary outcomes (composite of preterm birth and/or low birth weight, and prevalence of maternal CT/NG infection at delivery) will be assessed using intention to treat analysis at the clinic level. We will conduct both unadjusted and adjusted analyses, including risk factors for maternal STI and neonatal infection that may be imbalanced between the testing and standard-of-care clinics. Effect measures will be estimated with 95% confidence intervals. All analyses will be carried out using the latest version of Stata statistical software (17.0 or higher; College Station, TX, USA).

To assess immune/inflammatory markers, multiple logistic regression models will be developed in an iterative process to identify individual cytokines and groups of cytokines associated with mother-to-child transmission of CT/NG, preterm birth, and low birth weight. Additionally, we will identify plasma cytokine signatures that classify study participants with or without preterm birth/low birth weight infants [81]. We will define three equal frequency tertiles or “bins” (low, medium, and high), using all plasma samples. Next, we will use the *VSURF* R package, a random forest method to select the most predictive cytokines to differentiate samples from participants with and without preterm birth/low birth weight infants [82]. From these, we will build a decision tree to classify the samples. To better calibrate the accuracy of the decision tree, we will perform permutation testing.

To estimate the budget impact of CT/NG testing among asymptomatic pregnant women, we will combine the total recurrent costs with capital, training, and start-up costs. Next, we will build a decision analytic model to estimate the cost per pregnant woman tested, tested positive, treated, and cured by time of delivery. If the intervention is effective, we will calculate the incremental costs per adverse neonatal outcome averted (e.g. neonatal CT/NG infections, preterm birth, and low birth weight) comparing the testing intervention with syndromic management. We will also estimate a cost-effectiveness ratio (ICER, difference in costs divided by difference in health outcomes), representing the change in costs per averted DALYs associated with preterm birth/low birth weight infants.

We will also perform a probabilistic uncertainty analysis where parameters are randomly sampled from probabilistic distributions to generate 10,000 parameter sets. For each parameter set, the model will run and produce a distribution of outcomes. We will provide the means and 95% confidence intervals for all cost and health outcomes. If the intervention is effective, we will also calculate mean incremental cost-effectiveness ratios (ICER; $/DALY averted) compared to syndromic management, assessing cost-effectiveness under a willingness-to-pay threshold informed by the WHO’s Gross Domestic Product/capita threshold, which was US$7,961 in Botswana 2019. We will also calculate the probability that the scenarios are cost-effective by assessing the proportion of ICERs that fall below the willingness to pay threshold [83].

Sensitivity analyses will be conducted through one-way and multi-way methods to identify key cost drivers and assess how changing parameters impacts the overall costs and per participant cost of each strategy [21].

### Sample size

The sample size of 500 participants was determined to have sufficient power to detect a difference in rates of vertical transmission of CT and NG based on (1) the prevalence of CT and/or NG among pregnant women in Botswana (10%) [80], and (2) the risk for vertical transmission of CT/NG to newborn infants during parturition (50%).[44–47]. Antenatal CT/NG testing and treatment is expected to reduce vertical transmission by over 85%, from 5% in the control to 0.7% in the intervention group.[44, 45] When accounting for a 7% loss to follow-up rate, 250 women per arm will yield N = 232 evaluable subjects. With N = 232 evaluable samples per arm, we achieve 80.7% power to detect a 4.3% absolute difference in our primary endpoint, which is the proportion of vertical transmission of CT/NG, between the two groups, using a one-sided Fisher’s Exact test, at a 0.05 significance level.

### Methods for minimizing bias

Enrollment statistics, including total number of women attending their first antenatal clinic visit, total enrolled, total ineligible, and total who declined to participate, will be recorded in a weekly tally sheet and presented at bimonthly study meetings. Participant characteristics, such as sociodemographic characteristics, gestational age, HIV infection status, syphilis test results, obstetric history (e.g. history of preterm birth), in the testing and standard-of-care clinics will be monitored and compared to assess balance. Attrition will also be monitored and attrition bias will be assessed on a monthly bias.

### Data management

Data will be collected and managed using REDCap electronic data capture tools hosted at the Botswana Harvard AIDS Institute Partnership (BHP). REDCap (Research Electronic Data Capture) is a secure, web-based software platform designed to support data capture for research studies, providing (1) an intuitive interface for validated data capture; (2) audit trails for tracking data manipulation and export procedures; (3) automated export procedures for seamless data downloads to common statistical packages; and (4) procedures for data integration and interoperability with external sources.

Data will be entered offline at study clinics into CRFs on the REDCap mobile application on study tablets. At the end of each working day, data will be checked for any errors, discrepancies and missing data and will be uploaded to the main server by connecting to the internet. The BHP REDCap server is backed up daily. Backups are sent to a remote server and are also stored on tape. The IT System Administrator is responsible for monitoring backups.

Linking forms that link the participant’s study ID with their name and contact details will be recorded on separate hard copy linking forms and will be stored securely in a different location to the electronic data.

### Limitations

This study has several potential limitations. First, selection bias could be introduced if the populations in the testing and control clinics differ on important characteristics. For example, if women in the testing group have greater baseline STI prevalence, then the intervention will appear less effective at reducing neonatal infections. We believe that the inclusion of up to eight clinics in different parts of Gaborone will help balance participant characteristics between the two groups. Our study could also face attrition bias. We have devoted considerable study resources for follow-up staff, including a follow-up manager who will lead a team focused on retaining participants. We will also provide reimbursements to participants to cover transportation costs.

## Discussion

If the Maduo study is found to be effective, participants in the testing group will be treated for asymptomatic CT and NG infections that would have been missed through syndromic management and will have a lower risk for having an infant infected with CT and/or NG infection. Further, results from this study could encourage expansion of STI screening during antenatal care in low resource settings and improve maternal and neonatal health globally.

## Data Availability

All data underlying the results are available as part of the article and no additional source data are required.

## Abbreviations

ANC: Antenatal care
ART: Antiretroviral therapy
BHP: Botswana Harvard AIDS Institute Partnership
CDC: Centers for Disease Control and Prevention
CT: *Chlamydia trachomatis*
Ct: Cycle threshold value
EPT: Expedited partner therapy
ICER: Incremental cost effectiveness ratio
LMIC: Low and middle-income countries
NG: *Neisseria gonorrhoeae*
REDCap: Research Electronic Data Capture
SAC: Sample Adequacy Control
STI: Sexually transmitted infection
TAM: Time and motion study
WHO: World Health Organization
